# Variability of Steroid Response Time in Polymyalgia Rheumatica: A Case Report

**DOI:** 10.7759/cureus.31159

**Published:** 2022-11-06

**Authors:** Carter Gay, Colby Kihara, Katie Oakley, Arsh N Patel, O.P. Akinsoto

**Affiliations:** 1 Medicine, Alabama College of Osteopathic Medicine, Dothan, USA; 2 Internal Medicine, Athens Limestone Hospital, Athens, USA

**Keywords:** inflammatory arthritis, pelvic girdle, shoulders, muscle pain, low dose corticosteroids, polymyalgia rheumatica

## Abstract

We present a unique case of a 75-year-old Caucasian female who presented with a two-month history of unrelenting proximal muscle pain and stiffness in the neck, shoulders, and pelvic girdle that lasted for 45 minutes each morning upon waking. Due to clinical suspicion of polymyalgia rheumatica (PMR), the patient was started on the standard therapy of low-dose glucocorticoid therapy and was noted to have a dramatic improvement in terms of pain, strength, mobility, and range of motion. Current literature shows high variability in the standard response time to treatment. Typical resolution of symptoms occurs within a span of one day to months. The case presented in our study shows symptom resolution as well as marked improvement in muscle strength and mobility within 12 hours. The purpose of this case report is to provide additional information for physicians when considering symptom-resolution time related to low-dose glucocorticoid therapy and PMR. Additionally, we briefly explore the literature on the correlation between giant cell arteritis (GCA) and glucocorticoid therapy for PMR as well as the data associated with adjuvant therapy using immunomodulatory treatment.

## Introduction

Polymyalgia rheumatica (PMR) is an inflammatory rheumatic disease that characteristically occurs in individuals over 50, with females being at particularly high risk. Disease identification is based on clinical presentation and exclusion of other inflammatory diseases which can prove challenging as there are no pathognomonic features. Generally, the presentation of PMR includes proximal muscle pain and stiffness in the neck, shoulders, and hip girdle that lasts for at least 45 minutes each morning over a time frame of at least two weeks. Although there are no specific laboratory tests for PMR, there is a strong association with elevated inflammatory markers (erythrocyte sedimentation rate (ESR) and C-reactive protein (CRP)) [1]. Classically, treatments have included long-term low-dose prednisone (dose range from 12.5 to 25 mg per day) [2]. A rapid response to steroid treatment is considered an important component of the clinical diagnosis, yet this is problematic as there is a lack of consistent evidence for the appropriate response time to treatment. Several studies suggest a clinical response should occur within a few days [3] to one week [4], while other data indicates inadequate resolution even after three to four weeks [5,6]. Here, we demonstrate a case of PMR that resolved within 12 hours after the administration of 20 mg prednisone, which suggests that “rapid” response to medication may be more variable than previously understood.

## Case presentation

History

A 75-year-old female presented to the emergency department with a two-month history of muscle weakness and myalgia. Two months prior, the patient presented to the hospital for a urinary tract infection (UTI) and was treated at that time with antibiotics in addition to a low-dose steroid regimen for an inflammatory musculoskeletal process of unknown etiology. After tapering off the steroid regimen, the patient gradually experienced marked difficulty with ambulating, getting out of bed in the morning, and dressing herself. Review of systems was positive for diffuse muscle aches and weakness but negative for headache, jaw claudication, blurred vision, joint swelling, fever, and weight loss. Past medical history included hypertension, chronic kidney disease, hyperlipidemia, and type 2 diabetes. Her medications consisted of carvedilol 6.25 mg once daily, atorvastatin 40 mg once daily, and metformin 500 mg twice daily. Family history was negative for rheumatic disease or inflammatory arthritis. 

Examination

Upon physical examination, the patient was exquisitely tender to palpation, particularly proximally along the shoulders and pelvic girdle. Strength was graded as 3/5 in the upper extremities bilaterally, 2/5 in the hip flexors, and 3/5 in the remaining lower extremities. No formal gait assessment was performed as the patient was unable to tolerate weight bearing. Neurologic assessment revealed normal reflexes with no gross or focal deficits. There was no evidence of a rash or lesions on the skin. No joint swelling, warmth, or deformities were noted. 

Diagnostic imaging

**Figure 1:**
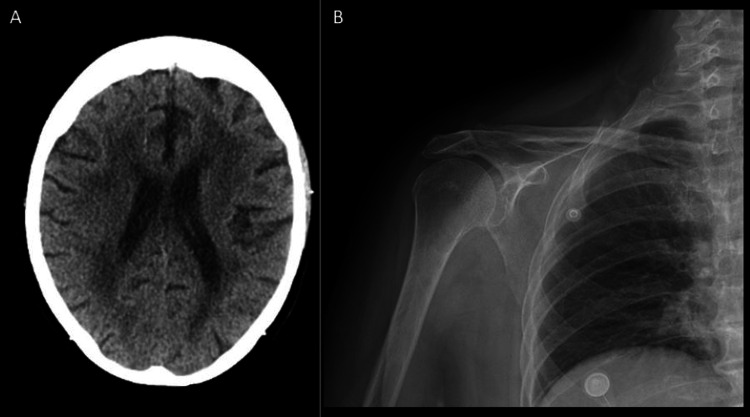
Radiologic imaging A) CT of the head revealed chronic findings without any acute intracranial abnormalities or neurovascular changes; B) X-ray of the right shoulder showed degenerative changes associated with mild osteoarthritis

A CT of the head was performed and shown as below in Figure 1. An X-ray of the right shoulder is also shown below in consideration of arthritic pathologies. 

Laboratory results

In consideration of PMR, ESR and CRP were elevated, which is consistent with diagnostic guidelines. Regarding myositis conditions and statin-related myopathy, creatine phosphokinase (CPK) was determined to be in normal range. Autoimmune assays were negative for anti-nuclear antibodies (ANA), rheumatoid factor, and anti-cyclic citrullinated peptide (CCP) antibodies. Other relevant findings are also displayed below in Table 1.

**Table 1:** Lab work and autoimmune assay results of the patient

Laboratory Results		
Labs	Patient Value	Reference Range
White blood cells (WBC)	6,800 cells/mm^3^	4,500-11,000 cells/mm^3^
Blood urea nitrogen (BUN)	29 mg/dL	8-21 mg/dL
Creatinine	1.5 mg/dL	0.7-1.3 mg/dL
Lactic acid	1.4 mmol/L	0.5-2.2 mmol/L
Erythrocyte sedimentation rate (ESR)	69 mm/hour	< 30 mm/hour
C-reactive protein (CRP)	12.6 mg/dL	0.0-0.5 mg/dL
Creatine phosphokinase (CPK)	56 U/L	24-173 U/L
Anti-nuclear antibodies (ANA)	Negative	
Rheumatoid factor	Negative	
Anti-cyclic citrullinated peptide (CCP) antibodies	Negative	

Treatment and outcome

The patient was started on a low-dose steroid regimen consisting of 20 mg prednisone daily. Within 12 hours of the first dose, the patient noted a marked improvement in her mobility and range of motion. Upon subsequent physical examination, the patient was nontender to palpation in her extremities and strength testing had improved to 4/5 in the upper and lower extremities bilaterally. Patient was able to ambulate with assistance of her walker and was stable for discharge the next day. CRP trended down from 12.6 mg/dL on admission to 2.2 mg/dL at discharge (normal: 0.0-0.5 mg/dL), while ESR remained elevated. Lastly, to mitigate the risk of long-term corticosteroid treatment, the patient was started on Vitamin D supplementation.

## Discussion

PMR is an inflammatory rheumatic disease that can have a severe impact on quality of life [5]. The diagnosis as well as the treatment of PMR hinges on the use of a low-dose steroid regimen, which may be problematic for a multitude of reasons. 

First, the lack of consistency in the time frame of response to steroid treatment makes it difficult to definitively conclude that the condition being treated is in fact PMR and that the response to therapy is appropriate. Literature provides support for improvement in muscle pain, stiffness and weakness occurring within several days, while other data indicates inadequate response occurring even after three to four weeks [3-6]. This contrast is highlighted by the dramatic response to treatment within 12 hours as documented in this report’s case presentation. Furthermore, the use of steroids may control symptoms and support the diagnosis of PMR, but this may also mask underlying inflammatory processes, as many conditions can have an initial or partial response to this steroid therapy. 

A well-documented problem with treatment of PMR is that the duration of therapy consistently lasts for one year or longer, with variable success rates in dose tapering and relapse rates as high as 50% upon discontinuation of steroids [7]. On the other hand, chronic use of steroids, albeit a low-dose regimen, presents a risk of adverse events including osteoporosis and bone fracture, which is concerning in elderly patients with underlying muscle pain and stiffness. Two retrospective studies examined the rates of adverse outcomes associated with long-term corticosteroid use in the treatment of PMR and determined that 65% of patients (81 of 124) and 43% of patients (95 of 222) experienced at least one adverse outcome, respectively [8,9]. 

Lastly, low-dose steroid treatment has not been shown to prevent progression to giant cell arteritis (GCA), a medical emergency that develops in up to 10% of individuals with PMR [4]. While muscular symptoms may be controlled, the patients remain at risk for complications, namely vision loss. Treatment of GCA classically requires a high-dose corticosteroid regimen, generally two to three times the dose required for PMR [10]. 

In terms of adjunctive treatments, data supporting methotrexate (MTX) is limited. There is evidence that MTX may allow faster tapering of steroid therapy in addition to a reduction of relapse rates, but this does not completely eliminate the use of long-term glucocorticoids [8]. 

A promising new treatment, tocilizumab, is a monoclonal interleukin 6 (IL-6) receptor blocker that has shown potential to treat PMR as well as GCA as monotherapy or as an adjunct. Elevated production of IL-6, a pro-inflammatory cytokine, is understood to be a key component in the pathogenesis of PMR and GCA [3].

## Conclusions

In the absence of proven alternatives, low-dose steroids remain a cornerstone in the management of PMR. Despite the many pitfalls of steroid therapy for PMR, the disease is unlikely to resolve without treatment and presents significant limitations on quality of life. Given the lack of consistency in the data, clinicians should not depend on the use of low-dose glucocorticoids and the response to therapy in the diagnosis and treatment of PMR. Providers should also be aware of the shortcomings and adverse considerations when using steroids in the management of PMR. 
